# Low-Power IMU System for Attitude Estimation-Based Plastic Greenhouse Foundation Uplift Monitoring

**DOI:** 10.3390/s25226901

**Published:** 2025-11-12

**Authors:** Gunhui Park, Junghwa Park, Eunji Jung, Jaehun Lee, Hyeonjun Hwang, Jisu Song, Seokcheol Yu, Seongyoon Lim, Jaesung Park

**Affiliations:** 1 Department of Bio-Industrial Machinery Engineering, College of Natural Resources and Life Science, Pusan National University, Miryang 50463, Republic of Korea; gunhui@pusan.ac.kr (G.P.); junghwa@pusan.ac.kr (J.P.); quberry9548@pusan.ac.kr (E.J.); wogns011025@pusan.ac.kr (J.L.); staccato43@pusan.ac.kr (H.H.); jiisu9380@pusan.ac.kr (J.S.); 2 Department of Agricultural Engineering, National Institute of Agricultural Sciences, Rural Development Administration, Jeonju 54875, Republic of Korea; seokcheol30@korea.kr (S.Y.); limsy73@korea.kr (S.L.); 3 Life and Industry Convergence Research Institute, Pusan National University, Miryang 50463, Republic of Korea

**Keywords:** plastic greenhouse, foundation uplift, structural health monitoring, inertial measurement unit, attitude estimation, LoRa, low-power wireless system, anomaly detection

## Abstract

Plastic greenhouses, which account for the majority of protected horticulture facilities in East Asia, are highly susceptible to wind-induced uplift failures that can lead to severe structural and economic damage. To address this issue, this study developed a low-power and low-cost wireless monitoring system applying the concept of structural health monitoring (SHM) to greenhouse foundations. Each sensor node integrates a MEMS-based inertial measurement unit (IMU) for attitude estimation, a LoRa module for long-range alert transmission, and a microSD module for data logging, while a gateway relays anomaly alerts to users through an IP network. Uplift tests were conducted on standard steel-pipe foundations commonly used in plastic greenhouses, and the proposed sensor nodes were evaluated alongside a commercial IMU to validate attitude estimation accuracy and anomaly detection performance. Despite the approximately 30-fold cost difference, comparable attitude estimation results were achieved. The system demonstrated low power consumption, confirming its feasibility for long-term operation using batteries or small solar cells. These results demonstrate the applicability of low-cost IMUs for real-time structural monitoring of lightweight greenhouse foundations.

## 1. Introduction

Plastic greenhouses play a pivotal role in East Asian agriculture. In South Korea, protected horticulture accounts for 11% of total agricultural production, and as of 2023, more than 99% of the total greenhouse area of 52,723 ha consists of plastic greenhouses [1]. In Japan, plastic greenhouses accounted for approximately 96% of the total greenhouse area of 40,590 ha in 2020 [2]. In China, the area of plastic greenhouses reached 1,183,877 ha in 2020, representing a 642.40% increase compared with 2000 [3]. Collectively, these figures highlight the central importance of plastic greenhouses as a production base in East Asian agriculture.

Plastic greenhouses are particularly vulnerable to wind loads. Uplift induced by strong winds can cause not only structural failure but also severe crop damage, leading to substantial recovery costs. In South Korea, between 2020 and 2023, approximately 73% of the economic losses to greenhouses from natural disasters were associated with wind-induced hazards, including strong winds, typhoons, and storm surges. During the same period, greenhouse damage accounted for about 25% of the total private property damage from these disasters [4].

To mitigate such losses, it is necessary to apply the concept of Structural Health Monitoring (SHM) to plastic greenhouses. SHM employs sensor networks to continuously monitor structural conditions, analyze collected data, diagnose damage, and assess stability [5]. To date, SHM has been extensively investigated in large civil structures such as bridges and dams [6], and has traditionally relied on uniaxial or triaxial accelerometers [7,8,9]. With the proliferation of low-cost microelectromechanical system (MEMS) accelerometers, research using these sensors has become increasingly active [10,11,12]. However, while expensive sensors are feasible for large-scale structures with high construction costs, their application to plastic greenhouses is economically impractical given the relatively low cost of these structures. Therefore, to apply SHM to plastic greenhouses, it is essential to use low-cost sensors such as MEMS-based inertial measurement units (IMUs).

IMUs provide measurements of acceleration and angular velocity, and optionally the magnetic field vector, relative to their intrinsic three-dimensional coordinate system [13]. In plastic greenhouses, structural tilt serves as a meaningful response indicator during foundation uplift. Structural tilt can be determined through attitude estimation, which can be performed using accelerometers and gyroscopes embedded in IMUs. Such attitude estimation mitigates the high-frequency noise and dynamic disturbances of accelerometers as well as the low-frequency drift of gyroscopes, thereby providing more accurate and robust attitude estimates of structural response than raw sensor signals. Research on IMU-based attitude estimation has been actively conducted in domains such as UAV and small satellite control [14,15,16], ship stabilization [17,18,19], and robotics [20,21,22]. Although IMUs have been applied to building tilt estimation [23,24], their application to lightweight agricultural structures, such as plastic greenhouses, remains largely unexplored.

Several practical requirements must be addressed to enable the effective monitoring of greenhouse foundations. Continuous data acquisition is necessary both before and after damage events for real-time alerts and post-disaster analyses. However, a commercial power supply cannot always be guaranteed during extreme weather events, and some greenhouses lack power facilities, necessitating the long-term operation of sensors with independent power sources. Moreover, stable data acquisition at a defined sampling interval is essential for IMU-based attitude estimation because the accuracy of the gyroscope-integrated attitude angles depends on the consistency of the sampling interval [25].

In the present study, a low-power and low-cost IMU-based monitoring system was developed to enable the long-term estimation and storage of greenhouse foundation attitudes and provide real-time anomaly alerts through a gateway in the event of foundation uplift. The system was evaluated through uplift tests on standard steel pipes (KS D 3760) used in plastic greenhouses, where it was deployed alongside a commercial IMU to determine suitable filters for accurate attitude estimation and validate the anomaly detection capability. The power consumption was estimated from the manufacturer’s datasheets and direct measurements of each module, and the feasibility of long-term data acquisition using battery power was evaluated.

## 2. Materials and Methods

### 2.1. Inertial Data Acquisition

A BMI160 IMU module (Bosch Sensortec, Reutlingen, Germany) was used to acquire inertial data. The BMI160 consists of a triaxial accelerometer and a triaxial gyroscope and operates at 3 V. The detailed specifications of the BMI160 are summarized in Table 1.

**Figure 1:**
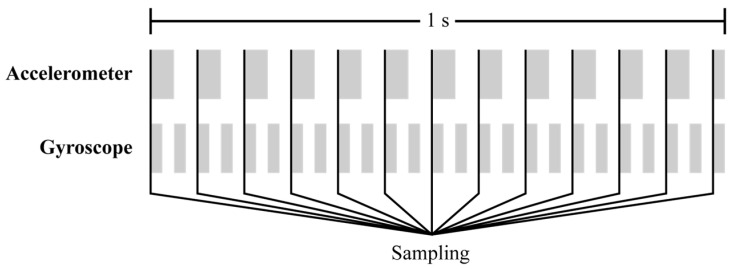
Synchronization and undersampling of the BMI160 accelerometer (12.5 Hz) and gyroscope (25 Hz) outputs over a 1 s period.

The output data rate (ODR) represents the sampling interval of a sensor; a higher ODR allows the detection of higher-frequency motions, but increases power consumption. For the BMI160, the accelerometer supports ODRs of 12.5–1600 Hz, and the gyroscope supports 25–3200 Hz. To capture the dynamic behavior of greenhouse foundations accurately, it is essential to configure an appropriate ODR considering the natural frequency of greenhouses. Choi et al. [26] conducted free-vibration tests on a single-span greenhouse and reported natural frequencies of 2.414 Hz and 4.339 Hz in the lateral and vertical directions, respectively. According to the Nyquist–Shannon sampling theorem, the sampling frequency must be at least twice the maximum input frequency to reconstruct the signal accurately. Based on this principle, the accelerometer and gyroscope were set to 12.5 Hz and 25 Hz, respectively, considering the allowable ODR ranges and ensuring that the sampling frequency exceeded twice the vertical natural frequency of the foundation of the single-span greenhouse. To synchronize the data acquisition rate, the gyroscope output was subsequently undersampled to 12.5 Hz. This synchronization and undersampling process is illustrated in Figure 1.

The BMI160 outputs two bytes of data per axis, yielding 12 bytes of acceleration and angular velocity data. To convert the raw outputs into physical quantities, a scale factor was applied, resulting in acceleration expressed in g and angular velocity in °/s. The BMI160 also contains an internal time counter with a resolution of 39 μs, stored in 3 bytes, which increments from 0 to 16,777,215 (0xFFFFFF) before overflowing and resetting to 0. Because the sensor node operates offline and cannot use an absolute time reference, such as Coordinated Universal Time (UTC), the elapsed time was calculated from successive measurements and converted into seconds. Consequently, the sensor node saves data at a rate of 12.5 Hz, consisting of time interval, x-axis acceleration, y-axis acceleration, z-axis acceleration, x-axis angular velocity, y-axis angular velocity and z-axis angular velocity.

### 2.2. Attitude Estimation

The pitch and yaw of the sensor node were computed using time intervals, along with the acceleration and angular velocity data [27,28]. The sensor node was assembled so that the x-axis of the IMU was oriented vertically. As a result, even when the node rotated in the roll direction, the gravity component remained unchanged, making roll estimation impossible. This is the same reason why commercial IMUs without a magnetometer cannot estimate yaw.

Attitude estimation is commonly performed using either a complementary or a Kalman filter [29]. The complementary filter is relatively simple to compute, whereas a general Kalman filter involves matrix operations based on a multivariable state–space model, and, thus, has a higher computational complexity [30,31]. Therefore, in this study, a first-order Kalman filter, which does not require matrix operations, was used.

The equations for calculating pitch (*θ*) and yaw (*ψ*) using the complementary filter are as follows:

(1) $$\theta_{k}^{\mathrm{acc}}=\tan^{-1}\left(\frac{a_{z,k}}{a_{x,k}}\right)\times \frac{180}{\pi }$$

(2) $$\psi_{k}^{\mathrm{acc}}=\tan^{-1}\left(\frac{-a_{y,k}}{a_{x,k}}\right)\times \frac{180}{\pi }$$

(3) $$\theta_{k}^{\mathrm{gyro}}=\theta_{k-1}+\omega_{y,k}\;\Delta t$$

(4) $$\psi_{k}^{\mathrm{gyro}}=\psi_{k-1}+\omega_{z,k}\;\Delta t$$

(5) $$\theta_{k}=\alpha \;\theta_{k}^{\mathrm{gyro}}+\left(1-\alpha \right)\;\theta_{k}^{\mathrm{acc}}$$

(6) $$\psi_{k}=\alpha \;\psi_{k}^{\mathrm{gyro}}+\left(1-\alpha \right)\;\psi_{k}^{\mathrm{acc}}$$

where *α* denotes the filter coefficient, *a* (g) the acceleration, *ω* (°/s) the angular velocity, and *t* (s) the time.

The equations for calculating pitch (*θ*) and yaw (*ψ*) using the first-order Kalman filter are given below, as $\theta_{k}^{\mathrm{acc}}$, $\psi_{k}^{\mathrm{acc}}$, $\theta_{k}^{\mathrm{gyro}}$ and $\psi_{k}^{\mathrm{gyro}}$ are obtained from Equations (1)–(4):

(7) $$P_{k}^{-}=P_{k-1}+Q$$

(8) $$K_{k}=\frac{P_{k}^{-}}{P_{k}^{-}+R}$$

(9) $$\theta_{k}=\theta_{k}^{\mathrm{gyro}}+K_{k}\left(\theta_{k}^{\mathrm{acc}}-\theta_{k}^{\mathrm{gyro}}\right)$$

(10) $$\psi_{k}=\psi_{k}^{\mathrm{gyro}}+K_{k}\left(\psi_{k}^{\mathrm{acc}}-\psi_{k}^{\mathrm{gyro}}\right)$$

(11) $$P_{k}=\left(1-K_{k}\right)\;P_{k}^{-}$$

where *Q* denotes the process noise covariance coefficient, *R* the measurement noise covariance coefficient.

### 2.3. Wireless Communication

For long-range wireless communication with locations equipped with an Internet Protocol (IP) network, a long-range (LoRa) module (E220-900T22D, Ebyte, Chengdu, China) based on an LLCC68 chipset (Semtech, Camarillo, CA, USA) was used, which enables stable transmission and reception over distances of up to 5 km. The detailed specifications of E220-900T22D are listed in Table 2.

The E220-900T22D module supports three operating modes. In normal mode, data transmission and reception are enabled, but the module consumes up to 110 mA during transmission. In contrast, sleep mode disables data communication but operates with a low current of approximately 5 μA. Accordingly, the transmitter was configured to switch to normal mode only during data transmission, while remaining in sleep mode to minimize power consumption. In contrast, the receiver was maintained in normal mode at all times to enable continuous data reception, as it is supplied with a stable power source.

**Figure 2:**
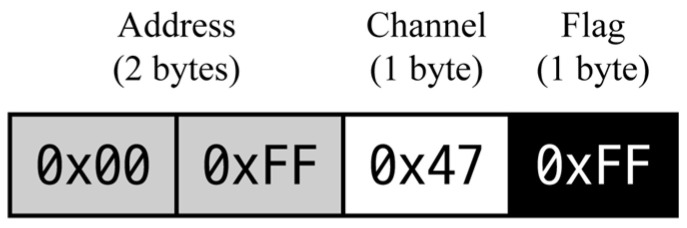
Transmission packet structure of the E220-900T22D.

The transmit power is a key factor influencing the communication range and is set to a maximum supported level of 22 dBm to enable long-distance transmission. To ensure communication reliability, each packet was configured to include the transmitter address and channel information, allowing reception by the intended receiver only. The structure of the transmission packet is shown in Figure 2. In addition, the Listen Before Talk (LBT) protocol was implemented to prevent collisions that may occur when multiple transmitters attempt to send data simultaneously. LBT detects channel occupancy prior to transmission and allows data to be sent only when the channel is clear, thereby minimizing the interference among multiple nodes sharing the same channel.

### 2.4. System Configuration

**Figure 3:**
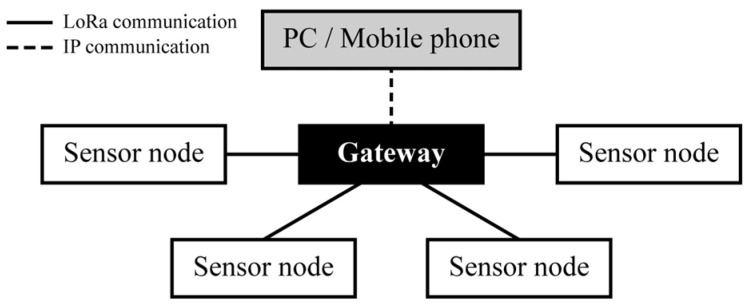
Star topology of the sensing system.

The sensing system is configured in a star topology consisting of sensor nodes and a gateway. The star topology of the sensing system is illustrated in Figure 3. The sensor nodes measured the inertial data of the greenhouse foundation, computed the attitude angles, and transmitted anomaly alerts, whereas the gateway received alerts transmitted from multiple sensor nodes and forwarded them to the user.

**Figure 4:**
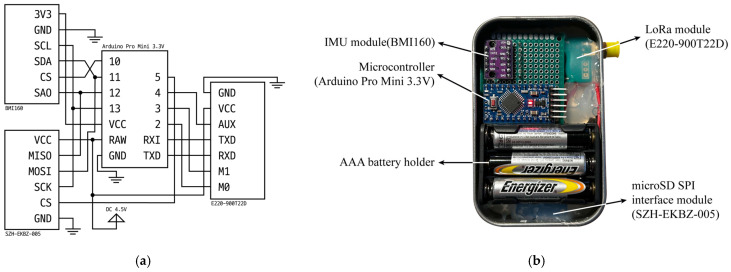
Circuit schematic and image of the sensor node: (**a**) Circuit schematic; (**b**) Image of the sensor node.

Each sensor node detects attitude changes using an embedded IMU and transmits a wireless packet to the gateway when the measured values exceed a predefined threshold. The gateway, which is installed in an environment with access to an IP network, receives data from the sensor nodes through long-range wireless communication. Because transmitting all samples to the gateway in real time inevitably leads to high power consumption, samples below the threshold were stored locally on the microSD card of the sensor mode in CSV format. A microSD SPI interface module (SZH-EKBZ-005, SZH) was used for the data storage. The sensor node comprises an ATmega328P-based microcontroller (Arduino Pro Mini, Arduino, Monza, Italy; Microchip Technology, Chandler, AZ, USA), IMU, LoRa module, and microSD module. Power was supplied by three 1.5 V batteries connected in series, providing 4.5 V. The supplied voltage was regulated to 3.3 V using an onboard regulator (MIC5205, Microchip Technology, Chandler, AZ, USA) and delivered to the IMU module, whereas the LoRa and microSD modules were directly powered at 4.5 V in parallel to ensure stable operation. The circuit schematic and hardware layout of the sensor node are shown in Figure 4.

**Figure 5:**
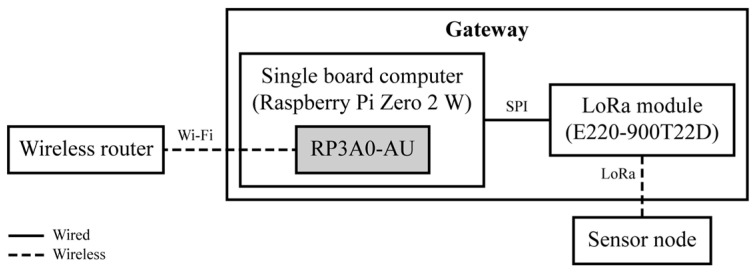
System diagram of the gateway.

The gateway consists of a single-board computer (Raspberry Pi Zero 2 W, Raspberry Pi Foundation, Cambridge, UK) and a LoRa module powered by a commercial power supply. The system diagram of the gateway is presented in Figure 5.

### 2.5. Field Test

#### 2.5.1. Case 1–2

**Figure 6:**
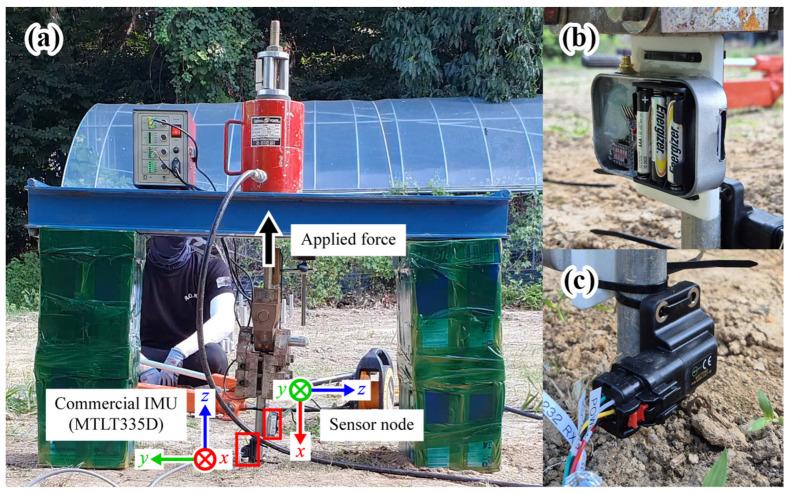
Mounting positions and coordinate systems for field test Cases 1–2: (**a**) Sensors; (**b**) Sensor node; (**c**) Commercial IMU.

To verify whether the uplift of plastic greenhouse foundations could be detected using inertial data, measurements were taken during a steel pipe uplift test. The test site was a paved area on the campus of University A (36°37′44″ N, 127°27′01″ E). Vertical hydraulic loads were incrementally increased by 20 kgf every 5 min for two steel pipes with different diameters: 48 mm (Case 1) and 25 mm (Case 2). Uplift was assumed to occur when a sudden drop in hydraulic pressure was observed, indicating that the applied load was no longer transmitted to the foundation. The hydraulic pressure values were monitored in real time through the testing machine display, and the exact moment of uplift was identified from the recorded video. For comparison with the developed sensor node, a commercial IMU (MTLT335D, ACEINNA, Tewksbury, MA, USA) commonly used in vehicles and construction machinery was installed. The sensor node mounting positions and coordinate systems for Cases 1–2 are presented in Figure 6.

#### 2.5.2. Case 3–4

To examine the behavior of the greenhouse foundation under normal conditions and to evaluate the operating stability of the sensor node, experiments were conducted in a single-span greenhouse located on the campus of University B (35°27′04″ N, 128°48′32″ E). In both cases, the sensor node was mounted on a 25 mm-diameter foundation steel pipe.

**Figure 7:**
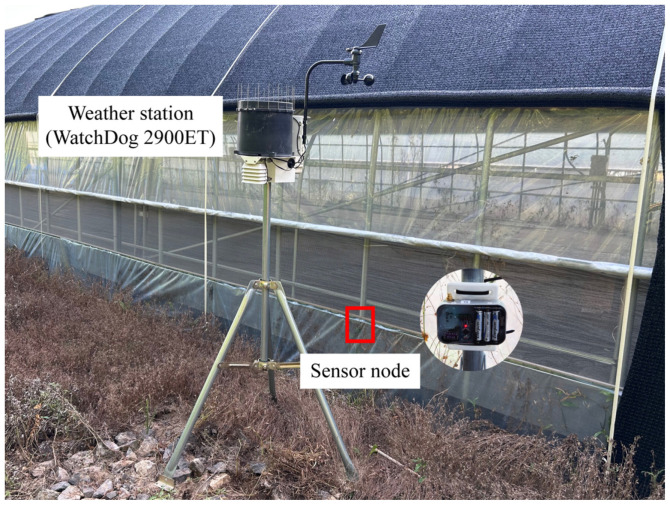
Installation location of the sensor node and the weather station for field test Case 3.

**Figure 8:**
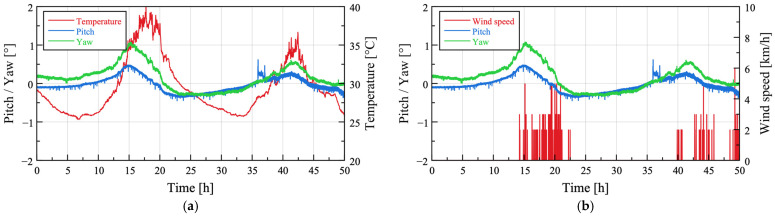
Relationships of attitude angles with temperature and wind speed during field test Case 3: (**a**) Temperature; (**b**) Wind speed.

For Case 3, data were collected for approximately 50 h, and a weather station (WatchDog 2900ET, Spectrum Technologies, Aurora, IL, USA) was installed to measure solar radiation, relative humidity, air temperature, rainfall, wind direction, wind gusts, wind speed, and dew point temperature. The installation location of the sensor node and the weather station for Case 3 are shown in Figure 7. Because IMUs exhibit variations in output with temperature changes, the segment between 22 and 24 h after installation, corresponding to a field temperature of 30–35 °C observed during the loading test at University A, was selected as the period representing normal conditions without external disturbances. Figure 8 presents the wind speed, temperature, and pitch and yaw values computed from the sensor node over the 50 h measurement period, with offsets applied relative to the initial values.

For Case 4, the sensor node was powered by three Li-FeS_2_ AAA batteries connected in series, and data was collected continuously for approximately 98 h until operation ceased. A summary of all field test cases and their conditions is provided in Table 3.

## 3. Results and Discussion

### 3.1. Comparison of Attitude Estimation Filters for the Sensor Node

Both complementary and Kalman filters were applied to the data collected from the sensor nodes to identify the filter type and coefficients that produced trends that were most similar to those of the commercial IMU. Owing to the installation orientation of the sensor node, only the pitch and yaw, which correspond to the roll and pitch of a commercial IMU, respectively, can be computed.

#### 3.1.1. Complementary Filter

**Figure 9:**
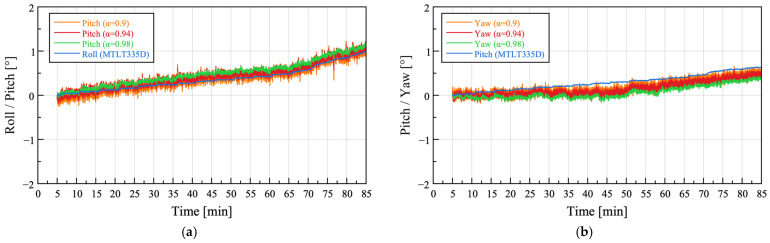

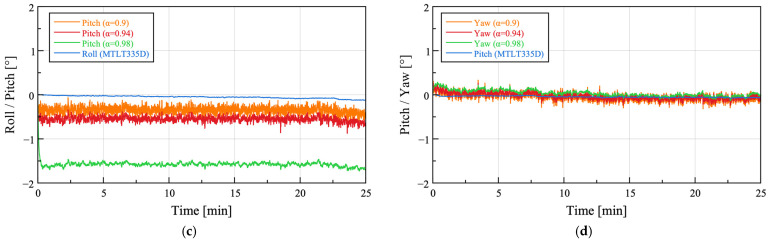
Attitude angle estimation using a complementary filter with varying *α* values: (**a**,**b**) Field test Case 1; (**c**,**d**) Field test Case 2.

The coefficient *α* of the complementary filter represents the reliability of the gyroscope-based estimate and is generally set between 0.9 and 1. For both Case 1 and Case 2, attitude angles were computed with *α* values of 0.9, 0.94, and 0.98, as shown in Figure 9. The outputs of both the commercial IMU and the sensor node were offset to zero at the initial value. Because the pitch of the commercial IMU and yaw of the sensor node are defined in opposite rotational directions, the yaw values of the sensor node were inverted for comparison.

To quantitatively evaluate the attitude characteristics of the sensor node according to variations in *α*, the root mean square error (RMSE) between the sensor node and the commercial IMU was calculated, and the standard deviation (SD) of the sensor node attitude angles was obtained to assess the noise level. Because the sampling rate of the sensor node was 12.5 Hz, whereas that of the commercial IMU was 10 Hz, the sensor node data were linearly interpolated to 10 Hz based on the timestamps of the commercial IMU to calculate the RMSE. The results are presented in Table 4.

In Case 1, the RMSE of the sensor node attitude angles increased as *α* increased, indicating a larger deviation from the commercial IMU. In Case 2, the RMSE of the sensor node pitch also tended to increase with *α*, showing that the RMSE at *α* = 0.98 was approximately three times greater than that at *α* = 0.94. Similarly, the yaw of the sensor node in Case 2 exhibited comparable RMSE values at *α* = 0.90 and 0.94, whereas the largest RMSE occurred at *α* = 0.98.

In contrast, the SD of the sensor node attitude angles showed negligible differences across *α* values. In Case 1, the ratio of the maximum to minimum SD for the pitch was approximately 1.008, indicating an almost identical level of noise, with the smallest SD observed at *α* = 0.94. The yaw of the sensor node also exhibited a small SD variation, with a maximum/minimum ratio of approximately 1.20, and the smallest SD was again observed at *α* = 0.94. In Case 2, the yaw showed a similar trend, with a maximum/minimum ratio of approximately 1.16 and a slight decrease in SD as *α* increased.

Overall, the SD of the sensor node attitude angles varied within a narrow range of 0.8–20%, suggesting that noise changes with *α* were minimal. Considering the sharp increase in RMSE of the sensor node pitch at *α* = 0.98 in Case 2, *α* = 0.94 was selected as the most appropriate complementary filter coefficient, providing both low noise and small deviation from the commercial IMU.

#### 3.1.2. Kalman Filter

**Figure 10:**
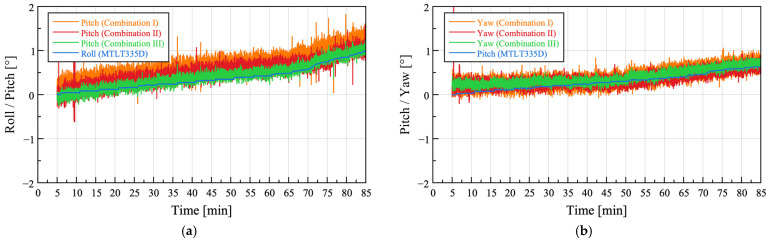

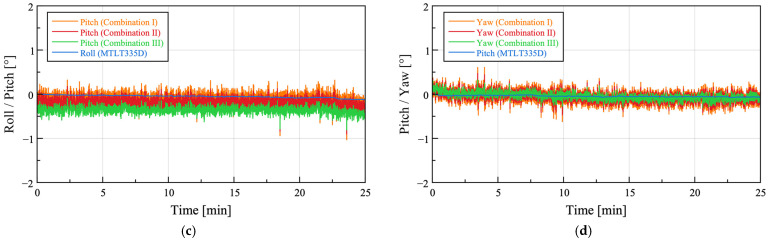
Attitude angle estimation using a Kalman filter with coefficient Combinations I–III: (**a**,**b**) Field test Case 1; (**c**,**d**) Field test Case 2.

As the process noise covariance coefficient *Q* increases, the prediction of the system model becomes less trustworthy, thereby increasing the contribution of the measurements. Conversely, as the measurement noise covariance coefficient *R* increases, the measurements become less trustworthy, thereby increasing the contribution of the predictions. Although *Q* and *R* can be adjusted individually for each sensor axis, the same values were used for simplification in this study. In general, the *Q* of a first-order Kalman filter is empirically tuned between 0.0001 and 0.01, whereas *R* is tuned between 0.01 and 1 depending on the characteristics of the system. In this study, three coefficient combinations were applied to Cases 1 and 2, and the results are presented in Figure 10. The *Q* and *R* coefficient combinations used in this study are summarized in Table 5. As with the complementary filter, the outputs are offset to zero at the initial value, and the yaw values of the sensor node are inverted.

To quantitatively evaluate the attitude characteristics of the sensor node according to variations in the Kalman filter coefficient combinations, the RMSE and SD were calculated in the same manner as for the complementary filter. The results are presented in Table 6.

In Case 1, the RMSE of the sensor node pitch decreased as *Q* decreased and *R* increased, whereas the yaw exhibited nearly identical RMSE values, with a maximum/minimum ratio of approximately 1.02. In Case 2, the pitch and yaw exhibited opposite trends, with the RMSE of the pitch increasing as *Q* decreased and *R* increased, whereas the RMSE of the yaw decreased under the same condition.

Regarding SD, the pitch and yaw in Case 1 showed little difference, with maximum/minimum ratios of approximately 1.07 and 1.15, respectively. In Case 2, both the pitch and yaw exhibited decreasing SD values as *Q* decreased and *R* increased.

Overall, Combination III was selected as the most appropriate Kalman filter coefficient combination, as it yielded both low RMSE and low SD values.

#### 3.1.3. Filter Selection

To evaluate which filter was more suitable for the system, the complementary filter with *α* = 0.94 and the Kalman filter with *Q* = 0.001 and *R* = 0.100 were compared based on RMSE and SD. The calculated RMSE and SD values used for comparison are listed in Table 7. In Case 2, the Kalman filter exhibited smaller RMSE values than the complementary filter for all axes except yaw. Even for the yaw, the RMSE of the Kalman filter was approximately 1.1 times that of the complementary filter, indicating only a minor difference. In particular, for the pitch in Case 2, the complementary filter showed an RMSE about 58% larger than that of the Kalman filter. Although the SD values of the Kalman filter were 6–17% higher than those of the complementary filter, the difference was considered negligible. Therefore, the Kalman filter, which achieved a larger RMSE improvement with comparable SD levels, was selected as the more suitable filter for this system.

### 3.2. Anomaly Detection of Greenhouse Foundations Using Attitude Angles

**Figure 11:**
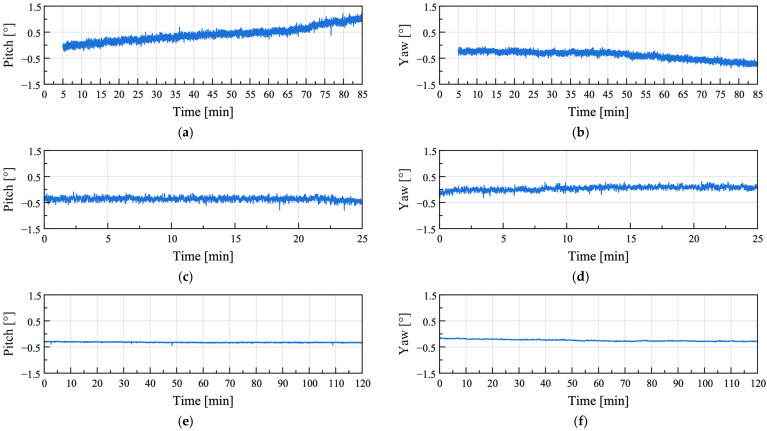
Attitude angles computed by the sensor node in field test Cases 1–3: (**a**,**b**) Field test Case 1; (**c**,**d**) Field test Case 2; (**e**,**f**) Field test Case 3.

To evaluate the feasibility of detecting anomalies in greenhouse foundations based on attitude angle variations, the attitude angles obtained from the sensor nodes during the uplift tests (Cases 1 and 2) were compared with those obtained under the baseline condition with minimal external influence (Case 3). The attitude angles for Cases 1–3 are shown in Figure 11.

Compared with instances where external influences were minimized (Figure 11e,f), significantly larger amplitudes were observed under uplift conditions (Figure 11a–d). To quantitatively evaluate this difference, the attitude angle data for each condition were differentiated, and the root mean square (RMS) values were calculated, as summarized in Table 8. The pitch and yaw in Cases 1 and 2 exhibited RMS values more than seven times greater than those in Case 3, confirming that the attitude angle variations were substantially larger under the uplift conditions than under the baseline condition.

**Figure 12:**
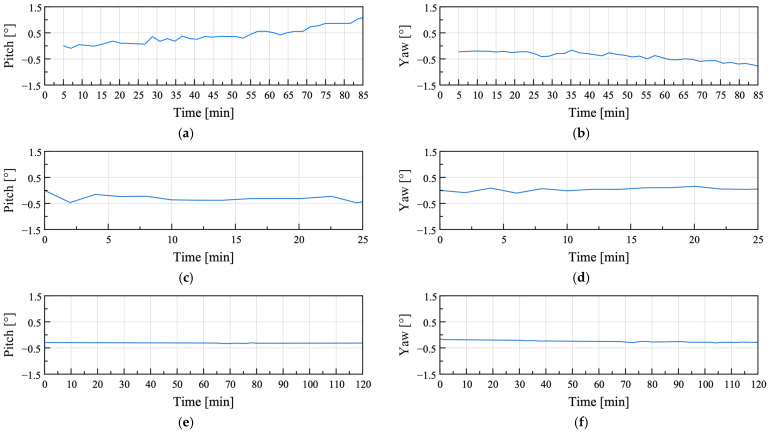
Attitude angles simplified using the Reumann–Witkam algorithm for field test Cases 1–3: (**a**,**b**) Field test Case 1; (**c**,**d**) Field test Case 2; (**e**,**f**) Field test Case 3.

To analyze the differences between the uplift and baseline conditions caused by gradual variations in attitude angles, the Reumann–Witkam algorithm was applied to linearize the attitude angle data. The Reumann–Witkam algorithm approximates points within a given tolerance by straight line segments, with larger tolerance values *ε* resulting in greater simplification of the curve. The results simplified with *ε* = 2 are presented in Figure 12.

**Figure 13:**
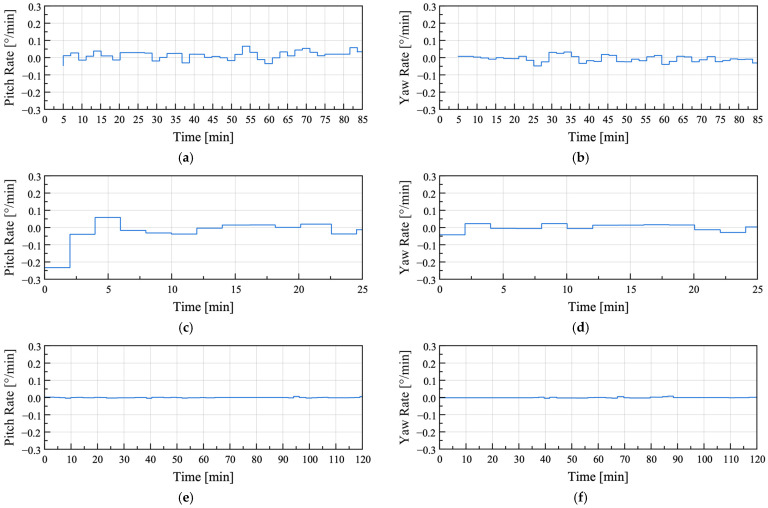
Rates of change in attitude angles simplified using the Reumann–Witkam algorithm for field test Cases 1–3: (**a**,**b**) Field test Case 1; (**c**,**d**) Field test Case 2; (**e**,**f**) Field test Case 3.

In the uplift condition shown in Figure 12a–d, continuous slope variations were observed over time, whereas in the baseline condition shown in Figure 12e,f, almost no slope variation occurred. The variations in the slope calculated for each condition were center-differentiated and are shown in Figure 13.

The maximum, minimum, and RMS values of the rate of change for each condition are summarized in Table 9. The pitch RMS of the baseline condition (Case 3) was approximately seven times smaller than that of Case 1 and approximately 24 times smaller than that of Case 2, whereas the yaw RMS was approximately 19 times smaller than that of Case 1 and approximately 11 times smaller than that of Case 2. These results indicate that the RMS values of the rate of change in the baseline condition were significantly lower than those in the uplift conditions.

### 3.3. Analysis of Power, Stability and Cost of the Sensor Node

#### 3.3.1. Power Consumption

To estimate the power consumption of the sensor nodes, the current consumption of each module was obtained from its respective datasheet. Although the rated current consumption of the Arduino Pro Mini is not specified by the manufacturer, user measurements indicated an average consumption of approximately 5 mA. At a supply voltage of 4.5 V, the power consumption was approximately 22.5 mW. The BMI160 IMU module consumes approximately 925 μA when both the accelerometer and gyroscope operate in normal mode, and with a supply voltage of 3.3 V from the Arduino Pro Mini, its power consumption is approximately 3.05 mW. The E220-900T22D LoRa module is normally maintained in sleep mode and communicates only when abnormal conditions are detected in the greenhouse structure. Accordingly, the sleep-mode current consumption was used for power estimation. The module consumes approximately 5 μA in sleep mode, and with a supply voltage of 4.5 V, its power consumption is approximately 0.023 mW. The microSD SPI interface module (SZH-EKBZ-005) consumes approximately 0.2 mA in idle mode, approximately 80 mA during typical read/write operations, and up to 200 mA at the maximum load. The sensor node performs data acquisition and logging at 800 ms intervals, of which the logging duration was measured using the Arduino micros() function to average approximately 0.6 ms. Thus, the remaining 799.4 ms can be regarded as idle time. Assuming an average current consumption of 100 mA during logging and 0.2 mA during idle time, the average power consumption of the micro-SD module was calculated as follows:

(12) $$\frac{0.2\times 799.4+100\times 0.6}{800}=0.27\;\mathrm{mW}$$

When the SZH-EKBZ-005 module was supplied at 4.5 V, its power consumption was approximately 1.22 mW. Consequently, the total power consumption of the sensor node is estimated to be approximately 26.81 mW. The estimated power consumption of sensor node components is summarized in Table 10.

To evaluate the power consumption under actual greenhouse foundation conditions, the consumption was calculated based on the battery capacity and operating time in Case 4. The batteries used (Energizer L92, Energizer, St. Louis, MO, USA) had a capacity of approximately 1200 mAh at a sensor node current consumption of approximately 6.2 mA. With the three batteries connected in series, the total supply voltage was 4.5 V, corresponding to a total stored energy of approximately 5.4 Wh. In Case 4, the sensor node operated for approximately 98 h, resulting in a power consumption of approximately 55.1 mW. This value is approximately twice the estimated 26.81 mW based on the datasheet specifications, likely owing to losses in the voltage regulator and discharge characteristics of the batteries. Nevertheless, the calculated power consumption corresponds to approximately 13.8% of the 400 mW consumed by the commercial IMU used in this study, revealing the relatively low power consumption of the sensor node.

#### 3.3.2. Operational Stability

**Figure 14:**
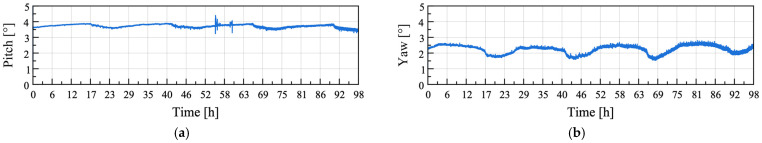
Attitude angles output from the sensor node in field test Case 4: (**a**) Pitch; (**b**) Yaw.

To evaluate the operational stability of the sensor node, the presence of missing data and the maintenance of the specified 80 ms data acquisition interval were examined in Case 4, where data were collected until battery depletion. The attitude angle outputs for Case 4 are presented in Figure 14. No missing data were detected using the R software (version 4.5.1) for analysis.

To achieve a data rate of 12.5 Hz, data must be stored every 80 ms. Measurements for which the elapsed time since the previous sample exceeded 82 ms were considered as outliers. The dataset was divided into 6 h intervals, and the outlier ratio for each interval (number of outliers/total number of samples in the interval) was examined. At all intervals, the outlier ratio remained at <1.1%, confirming stable data acquisition. The average sampling interval was 81.72 ms, with the first, median, and third quartiles all at 79.40 ms, and a maximum interval of 841.70 ms.

#### 3.3.3. Cost Analysis

To estimate the cost of the sensor node, the prices of individual components were obtained based on online retail prices. The prices of each component are listed in Table 11. The total cost of the sensor node was approximately USD 20, more than 20 times less expensive than the commercial IMU used for comparison.

## 4. Conclusions

In this study, a low-power and low-cost wireless monitoring system was developed by applying SHM technology to plastic greenhouses to detect the uplift behavior of the foundation and collect corresponding data. The system consists of sensor nodes that include an inertial measurement unit (IMU) for inertial data acquisition, a LoRa module for transmitting alerts when anomalies occur, a microSD module for data storage, and a gateway that receives alerts through the LoRa module and transmits them to users through an IP network.

The sensor nodes were evaluated by simultaneously collecting data with a commercial IMU during uplift tests on foundation steel pipes for plastic greenhouses, enabling the comparison and verification of the attitude estimation and anomaly detection performance. The RMSE values between the sensor nodes and the commercial IMU ranged from 0.06° to 0.32°, indicating that the sensor nodes achieved attitude estimation accuracy comparable to that of the commercial IMU despite a cost difference of approximately 20-fold. Furthermore, the analysis of attitude angle variations confirmed the feasibility of detecting uplift anomalies in greenhouse foundations.

An analysis of the power consumption of each module based on the manufacturer’s datasheets and direct measurements showed an average power consumption of approximately 55.1 mW, confirming the feasibility of long-term operation with batteries. This level of power consumption also falls within the range that can be sufficiently supplied through energy harvesting using small solar cells.

Low-cost IMUs have traditionally been applied in fields such as unmanned aerial vehicles (UAVs) and robotics, with limited applications in structural monitoring. Even in structural monitoring, their use has been largely restricted to large-scale structures such as bridges and high-rise buildings. This study showed the applicability of low-cost IMUs for monitoring lightweight structures such as plastic greenhouses. Recently, research on industrial automation and quality enhancement using machine learning and artificial intelligence (AI) has been actively conducted across various fields [32]. In particular, the integration of AI-based analysis with edge-computing architectures has been highlighted as a key element of smart manufacturing and autonomous monitoring systems. Applying these methodologies to the present system is expected to enable more active and accurate detection of foundation uplift by replacing the conventional threshold-based method with intelligent, data-driven algorithms. In future work, a large-scale collection of uplift data under diverse soil conditions and integration with edge-computing-based anomaly-detection algorithms are expected to further enhance the system into a real-time and high-precision structural anomaly-detection solution.

## Figures and Tables

**Table 1:** Specifications of BMI160.

Specification		Value			
		Min.	Typ.	Max.	Unit
General	Supply Voltage	1.71	3.0	3.6	V
	Current Consumption		925	990	μA
	Operating Temperature	−40		85	°C
	Photograph of the Module	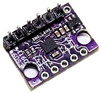			
Accelerometer	Measurement Range	±2/±4/±8/±16			g
	Resolution	16			bit
	Output Data Rate	12.5		1600	Hz
	Output Noise		180	300	μg/√Hz
Gyroscope	Measurement Range	±125/±250/±500/±1000/±2000			°/s
	Resolution	16			bit
	Output Data Rate	25		3200	Hz
	Output Noise		0.007		°/s/√Hz

**Table 2:** Specifications of E220-900T22D.

Specification	Value			
	Min.	Typ.	Max.	Unit
Operating Voltage	2.3	5	5.5	V
Operating Temperature	−40		85	°C
Operating Frequency	850.125		930.125	MHz
TX Power Consumption		110		mA
RX Power Consumption		16.8		mA
Sleep Power Consumption		5		μA
Maximum TX Power	21.5	22.0	22.5	dBm
Receiving Sensitivity	−146	−147	−148	dBm
Air Data Rate	2.4	2.4	62.5	kbps
Distance for Reference		5		km
Antenna	SMA-K			
Photograph of the Module	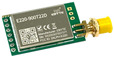			

**Table 3:** Field test cases and conditions.

Case	Test Conditions		Measurement Sensors		Test Sites
	Pipe Diameter	Test Duration	Sensor Node	Commercial IMU	
1	48 mm	85 min	Yes	Yes	36°37′44″ N 127°27′01″ E
2	25 mm	25 min	Yes	Yes	36°37′44″ N 127°27′01″ E
3	25 mm	50 h	Yes	No	35°27′04″ N 128°48′32″ E
4	25 mm	98 h	Yes	No	35°27′04″ N 128°48′32″ E

**Table 4:** RMSE and SD of the sensor node attitude angles for different *α* values.

Sensor Node Attitude Angle	Field Test Case	*α*	RMSE	SD
Pitch	1	0.90	0.204257	0.290637
		0.94	0.213562	0.288363
		0.98	0.235414	0.289325
	2	0.90	0.305509	0.077097
		0.94	0.506111	0.064308
		0.98	1.530756	0.072040
Yaw	1	0.90	0.193626	0.166899
		0.94	0.208040	0.160779
		0.98	0.267402	0.146637
	2	0.90	0.069746	0.078966
		0.94	0.061989	0.070438
		0.98	0.102013	0.067897

**Table 5:** Process and measurement noise covariance coefficients (*Q* and *R*) values for Kalman filter coefficient Combinations I–III.

Combination	Process Noise Covariance Coefficient (*Q*)	Measurement Noise Covariance Coefficient (*R*)
I	0.005	0.050
II	0.003	0.075
III	0.001	0.100

**Table 6:** RMSE and SD of the sensor node attitude angles for different coefficient combinations.

Sensor Node Attitude Angle	Field Test Case	Coefficient Combination	RMSE	SD
Pitch	1	I	0.365445	0.304221
		II	0.246787	0.283763
		III	0.204730	0.290319
	2	I	0.153176	0.122560
		II	0.184377	0.100206
		III	0.320618	0.075506
Yaw	1	I	0.205078	0.185625
		II	0.200235	0.161101
		III	0.204302	0.166256
	2	I	0.104382	0.113416
		II	0.087616	0.096016
		III	0.068740	0.078073

**Table 7:** RMSE and SD comparison of the sensor node attitude angles for complementary and Kalman filters.

Sensor Node Attitude Angle	Field Test Case	Complementary Filter (*α* = 0.94)		Kalman Filter (*Q* = 0.001, *R* = 0.1)	
		RMSE	SD	RMSE	SD
Pitch	1	0.213562	0.288363	0.204730	0.290319
	2	0.506111	0.064308	0.320618	0.075506
Yaw	1	0.208040	0.160779	0.204302	0.166256
	2	0.061989	0.070438	0.068740	0.078073

**Table 8:** RMS of differentiated attitude angles for field test Cases 1–3.

Field Test Case	Pitch	Yaw
1	0.0280	0.0227
2	0.0290	0.0250
3	0.00405	0.00275

**Table 9:** Maximum, minimum, and RMS values of the rate of change in attitude angles for field test Cases 1–3.

Field Test Case		Max.	Min.	RMS
1	Pitch	0.066336	−0.045503	0.0285
	Yaw	0.369066	−0.048114	0.0601
2	Pitch	0.254400	−0.233007	0.0908
	Yaw	0.022610	−0.106365	0.0334
3	Pitch	0.019826	−0.004367	0.00386
	Yaw	0.007690	−0.004772	0.00309

**Table 10:** Estimated power consumption of the sensor node components.

Components	Remarks	Power Consumption [mW]
Arduino Pro Mini 3.3 V		22.5
BMI160		3.05
E220-900T22D	Sleep Mode	0.023
SZH-EKBZ-005	Read/Write Mean	1.22
Total		26.81

**Table 11:** Estimated cost of the sensor node components.

Components	Cost [USD]
Arduino Pro Mini 3.3 V	11
BMI160	2
E220-900T22D	6
SZH-EKBZ-005	1
Total	20

## Data Availability

The data used to support the findings of this study are available from the corresponding author upon request.
